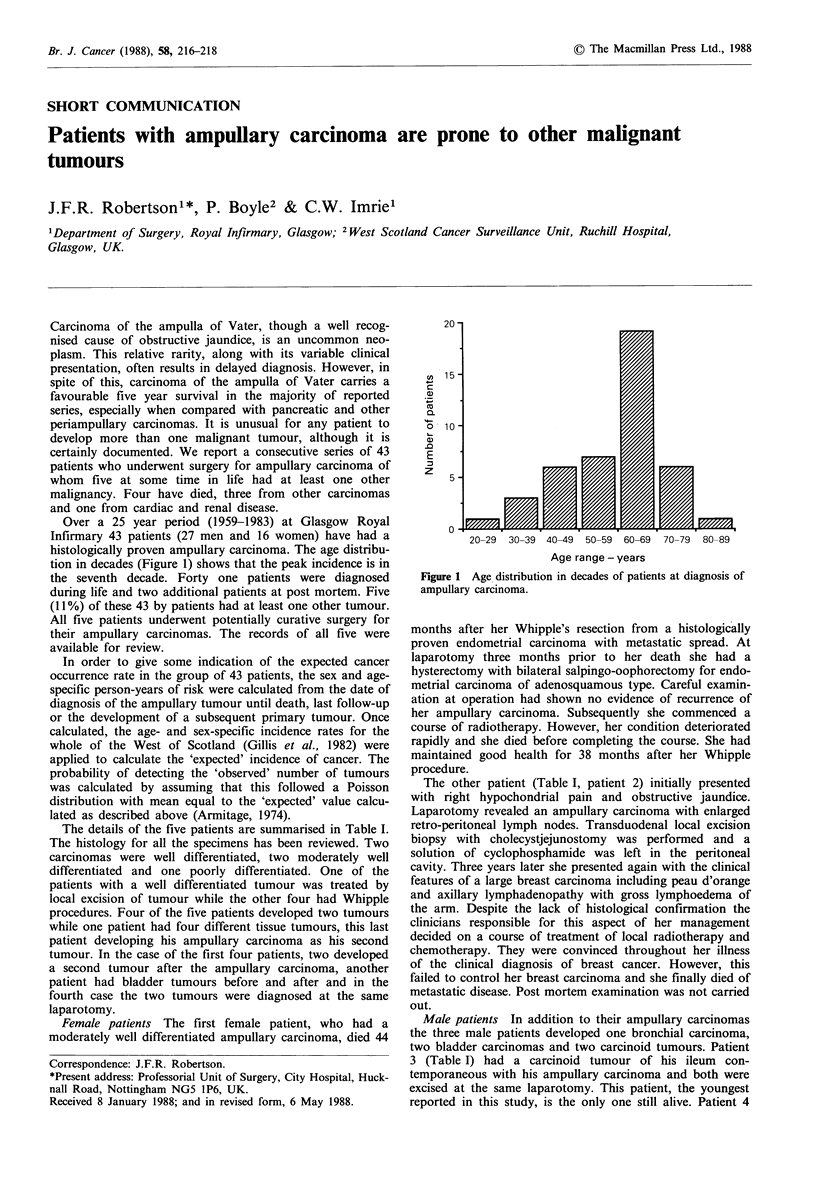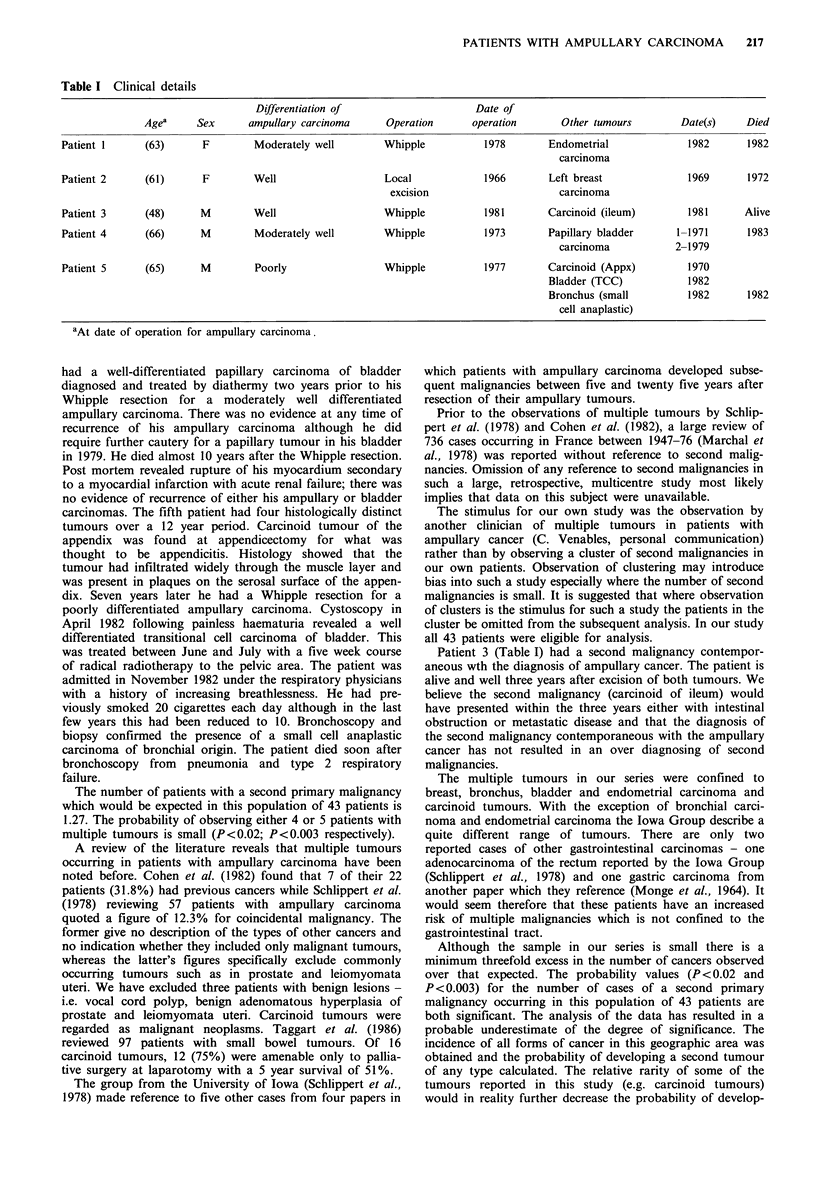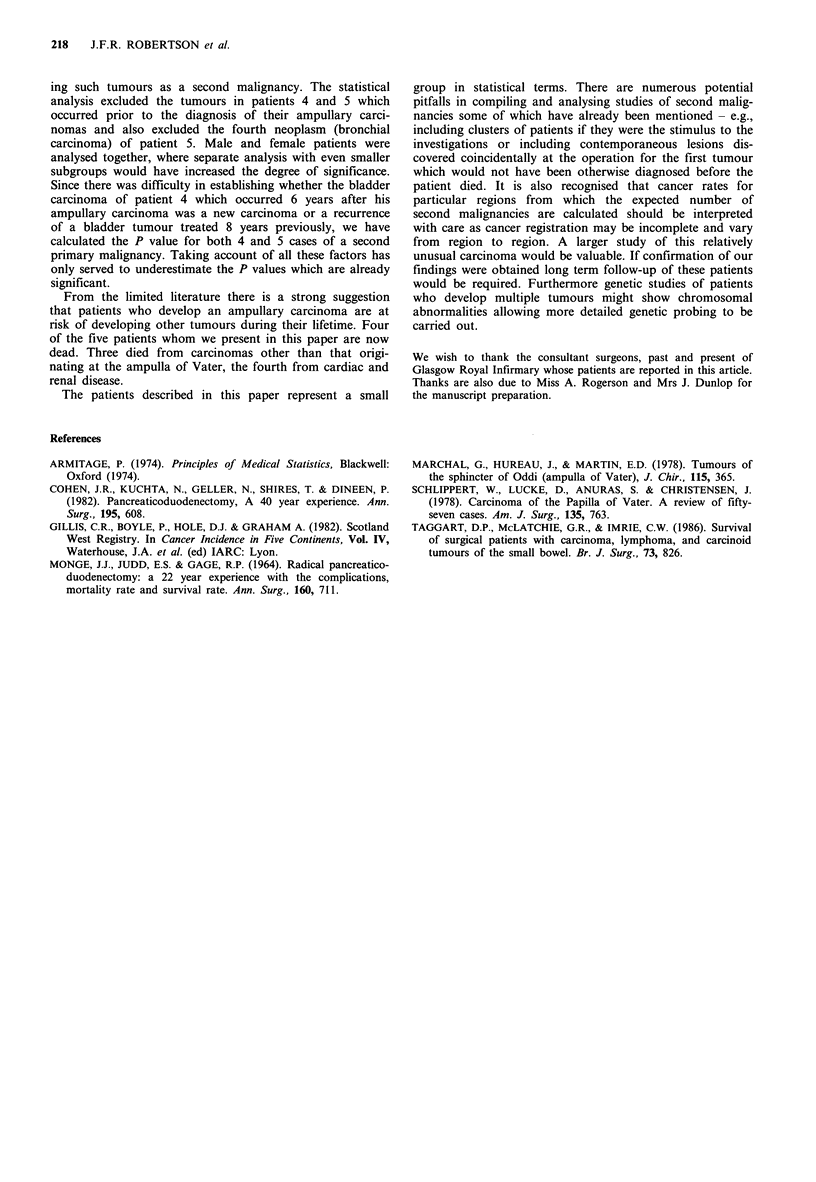# Patients with ampullary carcinoma are prone to other malignant tumours.

**DOI:** 10.1038/bjc.1988.197

**Published:** 1988-08

**Authors:** J. F. Robertson, P. Boyle, C. W. Imrie

**Affiliations:** Department of Surgery, Royal Infirmary, Glasgow, UK.


Br. J. Cancer (1988), 58, 216–218 © The Macmillan Press Ltd., 1988

SHORT COMMUNICATION

### Patients with ampullary carcinoma are prone to other malignant tumours

J.F.R. Robertson[1]*, P. Boyle[2] & C.W. Imrie[1]

[1]*Department of Surgery, Royal Infirmary, Glasgow;* [2]*West Scotland Cancer Surveillance Unit, Ruchill Hospital, Glasgow, UK.*

Carcinoma of the ampulla of Vater, though a well recognised cause of obstructive jaundice, is an uncommon neoplasm. This relative rarity, along with its variable clinical presentation, often results in delayed diagnosis. However, in spite of this, carcinoma of the ampulla of Vater carries a favourable five year survival in the majority of reported series, especially when compared with pancreatic and other periampullary carcinomas. It is unusual for any patient to develop more than one malignant tumour, although it is certainly documented. We report a consecutive series of 43 patients who underwent surgery for ampullary carcinoma of whom five at some time in life had at least one other malignancy. Four have died, three from other carcinomas and one from cardiac and renal disease.

Over a 25 year period (1959–1983) at Glasgow Royal Infirmary 43 patients (27 men and 16 women) have had a histologically proven ampullary carcinoma. The age distribution in decades (Figure 1) shows that the peak incidence is in the seventh decade. Forty one patients were diagnosed during life and two additional patients at post mortem. Five (11%) of these 43 by patients had at least one other tumour. All five patients underwent potentially curative surgery for their ampullary carcinomas. The records of all five were available for review.

In order to give some indication of the expected cancer occurrence rate in the group of 43 patients, the sex and age-specific person-years of risk were calculated from the date of diagnosis of the ampullary tumour until death, last follow-up or the development of a subsequent primary tumour. Once calculated, the age- and sex-specific incidence rates for the whole of the West of Scotland (Gillis *et al.*, 1982) were applied to calculate the 'expected' incidence of cancer. The probability of detecting the 'observed' number of tumours was calculated by assuming that this followed a Poisson distribution with mean equal to the 'expected' value calculated as described above (Armitage, 1974).

The details of the five patients are summarised in Table I. The histology for all the specimens has been reviewed. Two carcinomas were well differentiated, two moderately well differentiated and one poorly differentiated. One of the patients with a well differentiated tumour was treated by local excision of tumour while the other four had Whipple procedures. Four of the five patients developed two tumours while one patient had four different tissue tumours, this last patient developing his ampullary carcinoma as his second tumour. In the case of the first four patients, two developed a second tumour after the ampullary carcinoma, another patient had bladder tumours before and after and in the fourth case the two tumours were diagnosed at the same laparotomy.

*Female patients* The first female patient, who had a moderately well differentiated ampullary carcinoma, died 44

**Figure 1** Age distribution in decades of patients at diagnosis of ampullary carcinoma.

months after her Whipple's resection from a histologically proven endometrial carcinoma with metastatic spread. At laparotomy three months prior to her death she had a hysterectomy with bilateral salpingo-oophorectomy for endometrial carcinoma of adenosquamous type. Careful examination at operation had shown no evidence of recurrence of her ampullary carcinoma. Subsequently she commenced a course of radiotherapy. However, her condition deteriorated rapidly and she died before completing the course. She had maintained good health for 38 months after her Whipple procedure.

The other patient (Table I, patient 2) initially presented with right hypochondrial pain and obstructive jaundice. Laparotomy revealed an ampullary carcinoma with enlarged retro-peritoneal lymph nodes. Transduodenal local excision biopsy with cholecystjejunostomy was performed and a solution of cyclophosphamide was left in the peritoneal cavity. Three years later she presented again with the clinical features of a large breast carcinoma including peau d'orange and axillary lymphadenopathy with gross lymphoedema of the arm. Despite the lack of histological confirmation the clinicians responsible for this aspect of her management decided on a course of treatment of local radiotherapy and chemotherapy. They were convinced throughout her illness of the clinical diagnosis of breast cancer. However, this failed to control her breast carcinoma and she finally died of metastatic disease. Post mortem examination was not carried out.

*Male patients* In addition to their ampullary carcinomas the three male patients developed one bronchial carcinoma, two bladder carcinomas and two carcinoid tumours. Patient 3 (Table I) had a carcinoid tumour of his ileum contemporaneous with his ampullary carcinoma and both were excised at the same laparotomy. This patient, the youngest reported in this study, is the only one still alive. Patient 4

Correspondence: J.F.R. Robertson.
*Present address: Professorial Unit of Surgery, City Hospital, Hucknall Road, Nottingham NG5 1P6, UK.
Received 8 January 1988; and in revised form, 6 May 1988.

**Table I** Clinical details

| | *Age*[a] | *Sex* | *Differentiation of ampullary carcinoma* | *Operation* | *Date of operation* | *Other tumours* | *Date(s)* | *Died* |
|---|---|---|---|---|---|---|---|---|
| Patient 1 | (63) | F | Moderately well | Whipple | 1978 | Endometrial carcinoma | 1982 | 1982 |
| Patient 2 | (61) | F | Well | Local excision | 1966 | Left breast carcinoma | 1969 | 1972 |
| Patient 3 | (48) | M | Well | Whipple | 1981 | Carcinoid (ileum) | 1981 | Alive |
| Patient 4 | (66) | M | Moderately well | Whipple | 1973 | Papillary bladder carcinoma | 1–1971<br>2–1979 | 1983 |
| Patient 5 | (65) | M | Poorly | Whipple | 1977 | Carcinoid (Appx)<br>Bladder (TCC)<br>Bronchus (small cell anaplastic) | 1970<br>1982<br>1982 | <br><br>1982 |

[a]At date of operation for ampullary carcinoma.

had a well-differentiated papillary carcinoma of bladder diagnosed and treated by diathermy two years prior to his Whipple resection for a moderately well differentiated ampullary carcinoma. There was no evidence at any time of recurrence of his ampullary carcinoma although he did require further cautery for a papillary tumour in his bladder in 1979. He died almost 10 years after the Whipple resection. Post mortem revealed rupture of his myocardium secondary to a myocardial infarction with acute renal failure; there was no evidence of recurrence of either his ampullary or bladder carcinomas. The fifth patient had four histologically distinct tumours over a 12 year period. Carcinoid tumour of the appendix was found at appendicectomy for what was thought to be appendicitis. Histology showed that the tumour had infiltrated widely through the muscle layer and was present in plaques on the serosal surface of the appendix. Seven years later he had a Whipple resection for a poorly differentiated ampullary carcinoma. Cystoscopy in April 1982 following painless haematuria revealed a well differentiated transitional cell carcinoma of bladder. This was treated between June and July with a five week course of radical radiotherapy to the pelvic area. The patient was admitted in November 1982 under the respiratory physicians with a history of increasing breathlessness. He had previously smoked 20 cigarettes each day although in the last few years this had been reduced to 10. Bronchoscopy and biopsy confirmed the presence of a small cell anaplastic carcinoma of bronchial origin. The patient died soon after bronchoscopy from pneumonia and type 2 respiratory failure.

The number of patients with a second primary malignancy which would be expected in this population of 43 patients is 1.27. The probability of observing either 4 or 5 patients with multiple tumours is small ($P<0.02$; $P<0.003$ respectively).

A review of the literature reveals that multiple tumours occurring in patients with ampullary carcinoma have been noted before. Cohen *et al.* (1982) found that 7 of their 22 patients (31.8%) had previous cancers while Schlippert *et al.* (1978) reviewing 57 patients with ampullary carcinoma quoted a figure of 12.3% for coincidental malignancy. The former give no description of the types of other cancers and no indication whether they included only malignant tumours, whereas the latter's figures specifically exclude commonly occurring tumours such as in prostate and leiomyomata uteri. We have excluded three patients with benign lesions – i.e. vocal cord polyp, benign adenomatous hyperplasia of prostate and leiomyomata uteri. Carcinoid tumours were regarded as malignant neoplasms. Taggart *et al.* (1986) reviewed 97 patients with small bowel tumours. Of 16 carcinoid tumours, 12 (75%) were amenable only to palliative surgery at laparotomy with a 5 year survival of 51%.

The group from the University of Iowa (Schlippert *et al.*, 1978) made reference to five other cases from four papers in which patients with ampullary carcinoma developed subsequent malignancies between five and twenty five years after resection of their ampullary tumours.

Prior to the observations of multiple tumours by Schlippert *et al.* (1978) and Cohen *et al.* (1982), a large review of 736 cases occurring in France between 1947–76 (Marchal *et al.*, 1978) was reported without reference to second malignancies. Omission of any reference to second malignancies in such a large, retrospective, multicentre study most likely implies that data on this subject were unavailable.

The stimulus for our own study was the observation by another clinician of multiple tumours in patients with ampullary cancer (C. Venables, personal communication) rather than by observing a cluster of second malignancies in our own patients. Observation of clustering may introduce bias into such a study especially where the number of second malignancies is small. It is suggested that where observation of clusters is the stimulus for such a study the patients in the cluster be omitted from the subsequent analysis. In our study all 43 patients were eligible for analysis.

Patient 3 (Table I) had a second malignancy contemporaneous wth the diagnosis of ampullary cancer. The patient is alive and well three years after excision of both tumours. We believe the second malignancy (carcinoid of ileum) would have presented within the three years either with intestinal obstruction or metastatic disease and that the diagnosis of the second malignancy contemporaneous with the ampullary cancer has not resulted in an over diagnosing of second malignancies.

The multiple tumours in our series were confined to breast, bronchus, bladder and endometrial carcinoma and carcinoid tumours. With the exception of bronchial carcinoma and endometrial carcinoma the Iowa Group describe a quite different range of tumours. There are only two reported cases of other gastrointestinal carcinomas – one adenocarcinoma of the rectum reported by the Iowa Group (Schlippert *et al.*, 1978) and one gastric carcinoma from another paper which they reference (Monge *et al.*, 1964). It would seem therefore that these patients have an increased risk of multiple malignancies which is not confined to the gastrointestinal tract.

Although the sample in our series is small there is a minimum threefold excess in the number of cancers observed over that expected. The probability values ($P<0.02$ and $P<0.003$) for the number of cases of a second primary malignancy occurring in this population of 43 patients are both significant. The analysis of the data has resulted in a probable underestimate of the degree of significance. The incidence of all forms of cancer in this geographic area was obtained and the probability of developing a second tumour of any type calculated. The relative rarity of some of the tumours reported in this study (e.g. carcinoid tumours) would in reality further decrease the probability of developing such tumours as a second malignancy. The statistical analysis excluded the tumours in patients 4 and 5 which occurred prior to the diagnosis of their ampullary carcinomas and also excluded the fourth neoplasm (bronchial carcinoma) of patient 5. Male and female patients were analysed together, where separate analysis with even smaller subgroups would have increased the degree of significance. Since there was difficulty in establishing whether the bladder carcinoma of patient 4 which occurred 6 years after his ampullary carcinoma was a new carcinoma or a recurrence of a bladder tumour treated 8 years previously, we have calculated the $P$ value for both 4 and 5 cases of a second primary malignancy. Taking account of all these factors has only served to underestimate the $P$ values which are already significant.

From the limited literature there is a strong suggestion that patients who develop an ampullary carcinoma are at risk of developing other tumours during their lifetime. Four of the five patients whom we present in this paper are now dead. Three died from carcinomas other than that originating at the ampulla of Vater, the fourth from cardiac and renal disease.

The patients described in this paper represent a small group in statistical terms. There are numerous potential pitfalls in compiling and analysing studies of second malignancies some of which have already been mentioned – e.g., including clusters of patients if they were the stimulus to the investigations or including contemporaneous lesions discovered coincidentally at the operation for the first tumour which would not have been otherwise diagnosed before the patient died. It is also recognised that cancer rates for particular regions from which the expected number of second malignancies are calculated should be interpreted with care as cancer registration may be incomplete and vary from region to region. A larger study of this relatively unusual carcinoma would be valuable. If confirmation of our findings were obtained long term follow-up of these patients would be required. Furthermore genetic studies of patients who develop multiple tumours might show chromosomal abnormalities allowing more detailed genetic probing to be carried out.

We wish to thank the consultant surgeons, past and present of Glasgow Royal Infirmary whose patients are reported in this article. Thanks are also due to Miss A. Rogerson and Mrs J. Dunlop for the manuscript preparation.

**References**

ARMITAGE, P. (1974). *Principles of Medical Statistics*, Blackwell: Oxford (1974).

COHEN, J.R., KUCHTA, N., GELLER, N., SHIRES, T. & DINEEN, P. (1982). Pancreaticoduodenectomy, A 40 year experience. *Ann. Surg.*, **195**, 608.

GILLIS, C.R., BOYLE, P., HOLE, D.J. & GRAHAM A. (1982). Scotland West Registry. In *Cancer Incidence in Five Continents*, **Vol. IV**, Waterhouse, J.A. *et al.* (ed) IARC: Lyon.

MONGE, J.J., JUDD, E.S. & GAGE, R.P. (1964). Radical pancreaticoduodenectomy: a 22 year experience with the complications, mortality rate and survival rate. *Ann. Surg.*, **160**, 711.

MARCHAL, G., HUREAU, J., & MARTIN, E.D. (1978). Tumours of the sphincter of Oddi (ampulla of Vater), *J. Chir.*, **115**, 365.

SCHLIPPERT, W., LUCKE, D., ANURAS, S. & CHRISTENSEN, J. (1978). Carcinoma of the Papilla of Vater. A review of fifty-seven cases. *Am. J. Surg.*, **135**, 763.

TAGGART, D.P., McLATCHIE, G.R., & IMRIE, C.W. (1986). Survival of surgical patients with carcinoma, lymphoma, and carcinoid tumours of the small bowel. *Br. J. Surg.*, **73**, 826.